# Two decades of three-dimensional movement data from adult female northern elephant seals

**DOI:** 10.1038/s41597-024-04084-4

**Published:** 2024-12-18

**Authors:** Daniel P. Costa, Rachel R. Holser, Theresa R. Keates, Taiki Adachi, Roxanne S. Beltran, Cory D. Champagne, Daniel E. Crocker, Arina B. Favilla, Melinda A. Fowler, Juan Pablo Gallo-Reynoso, Chandra Goetsch, Jason L. Hassrick, Luis A. Hückstädt, Jessica M. Kendall-Bar, Sarah S. Kienle, Carey E. Kuhn, Jennifer L. Maresh, Sara M. Maxwell, Birgitte I. McDonald, Elizabeth A. McHuron, Patricia A. Morris, Yasuhiko Naito, Logan J. Pallin, Sarah H. Peterson, Patrick W. Robinson, Samantha E. Simmons, Akinori Takahashi, Nicole M. Teuschel, Michael S. Tift, Yann Tremblay, Stella Villegas-Amtmann, Ken Yoda

**Affiliations:** 1 Institute of Marine Sciences, University of California Santa Cruz, California, 95064 USA; 2 Department of Ecology and Evolutionary Biology, University of California Santa Cruz, California, 95064 USA; 3 Department of Ocean Sciences, University of California Santa Cruz, California, 95064 USA; 4 National Institute of Polar Research, Tachikawa, Tokyo Japan; 5 Department of Biology, Sonoma State University, Rohnert Park, California 94928 USA; 6 Biology Department, Northeastern University, Oakland, CA 94610 USA; 7 Centro de Investigación en Alimentación y Desarrollo, Coordinación Guaymas, Guaymas, Mexico; 8 CSS, Inc., Fairfax, Virginia 22031 USA; 9 National Centers for Coastal Ocean Science, NOAA, Silver Spring, Maryland 20910 USA; 10 ICF, Jones and Stokes, Inc., 980 9th Street, Suite 1200, Sacramento, CA 95814 USA; 11 Centre for Ecology and Conservation, University of Exeter, Penryn Campus, Penryn, TR10 9FE UK; 12 Scripps Institution of Oceanography, University of California San Diego, San Diego, CA USA; 13 Department of Biology, Baylor University, Waco, TX USA; 14 Marine Mammal Laboratory, Alaska Fisheries Science Center, National Oceanic and Atmospheric Administration, Seattle, WA USA; 15 Department of Biology, West Chester University, 730 S Church St, West Chester, PA 19383 USA; 16 School of Interdisciplinary Arts and Sciences, University of Washington, Bothell Campus, Bothell, WA USA; 17 Moss Landing Marine Labs, San Jose State University, Moss Landing, California, 95039 USA; 18 Cooperative Institute for Climate, Ocean, and Ecosystem Studies, University of Washington, Seattle, WA 98105 USA; 19 UC Año Nuevo Natural Reserve, University of California, Santa Cruz, California, 95064 USA; 20 SMRU Consulting, Scottish Oceans Institute, University of St Andrews, St Andrews, UK; 21 Department of Biology and Marine Biology, University of North Carolina Wilmington, Wilmington, NC 28403 USA; 22 Institut de Recherche pour le Developpement, Marseille, France; 23 Graduate School of Environmental Studies, Nagoya University, Furo-cho, Chikusa-ku, Nagoya, 464-8601 Japan

**Keywords:** Animal migration, Marine biology, Animal behaviour

## Abstract

Northern elephant seals (*Mirounga angustirostris*) have been integral to the development and progress of biologging technology and movement data analysis, which continue to improve our understanding of this and other species. Adult female elephant seals at Año Nuevo Reserve and other colonies along the west coast of North America were tracked annually from 2004 to 2020, resulting in a total of 653 instrument deployments. This paper outlines the compilation and curation process of these high-resolution diving and location data, now accessible in two Dryad repositories. The code used for data processing alongside the corresponding workflow is available through GitHub and Zenodo. This data set represents 3,844,927 dives and 596,815 locations collected from 475 individual seals with 178 repeat samplings over 17 years. We anticipate that these data will stimulate further analysis and investigation into elephant seal biology and aid in developing new analytical approaches for large marine predators.

## Background & Summary

Long-term data sets are essential for monitoring fine-scale changes in animal movement, behavior, and phenology over time and provide critical insights into population-level processes^1–3^. Biologging data, where electronic instruments attached to an animal collect data on the individual and its environment, allow researchers to characterize the distribution and behavior of populations^1–9^. This enables the observation of key phenological events such as mortality^10,11^ and illness^12^. Such long-term datasets are also critical for understanding the effects of climate variability and climate change, as researchers can examine fine-scale changes in wild animals’ movement patterns as their habitat changes^4,13–22^. They allow big data applications^23–26^ and contribute disproportionately to developing national and international management and conservation policies^27^. While long-time series are essential for all the reasons above, they are challenging to collect and maintain and are thus quite rare and seldom publicly available.

Northern elephant seals (*Mirounga angustirostris*) are excellent research subjects for biologging studies because they are easily approached on land, exhibit high natal philopatry and survival rates, and have a large body size that enables them to carry one or more biologging instruments (hereafter, “tags”). Elephant seals are capital breeders that travel thousands of kilometers during two extended foraging trips at sea. Adult females then spend one to two months fasting during their breeding (Jan-Feb) and molting (Apr-Jun) seasons^1,28,29^. Their short foraging trip, post-breeding, occurs between February and May, and the long gestational foraging trip, post-molting, spans from June to January^28,30^. The existence of a breeding colony at Año Nuevo Reserve, 21 miles north of the UC Santa Cruz campus, has enabled an ongoing elephant seal research program that has spanned five decades^31,32^.

**Fig. 1:**
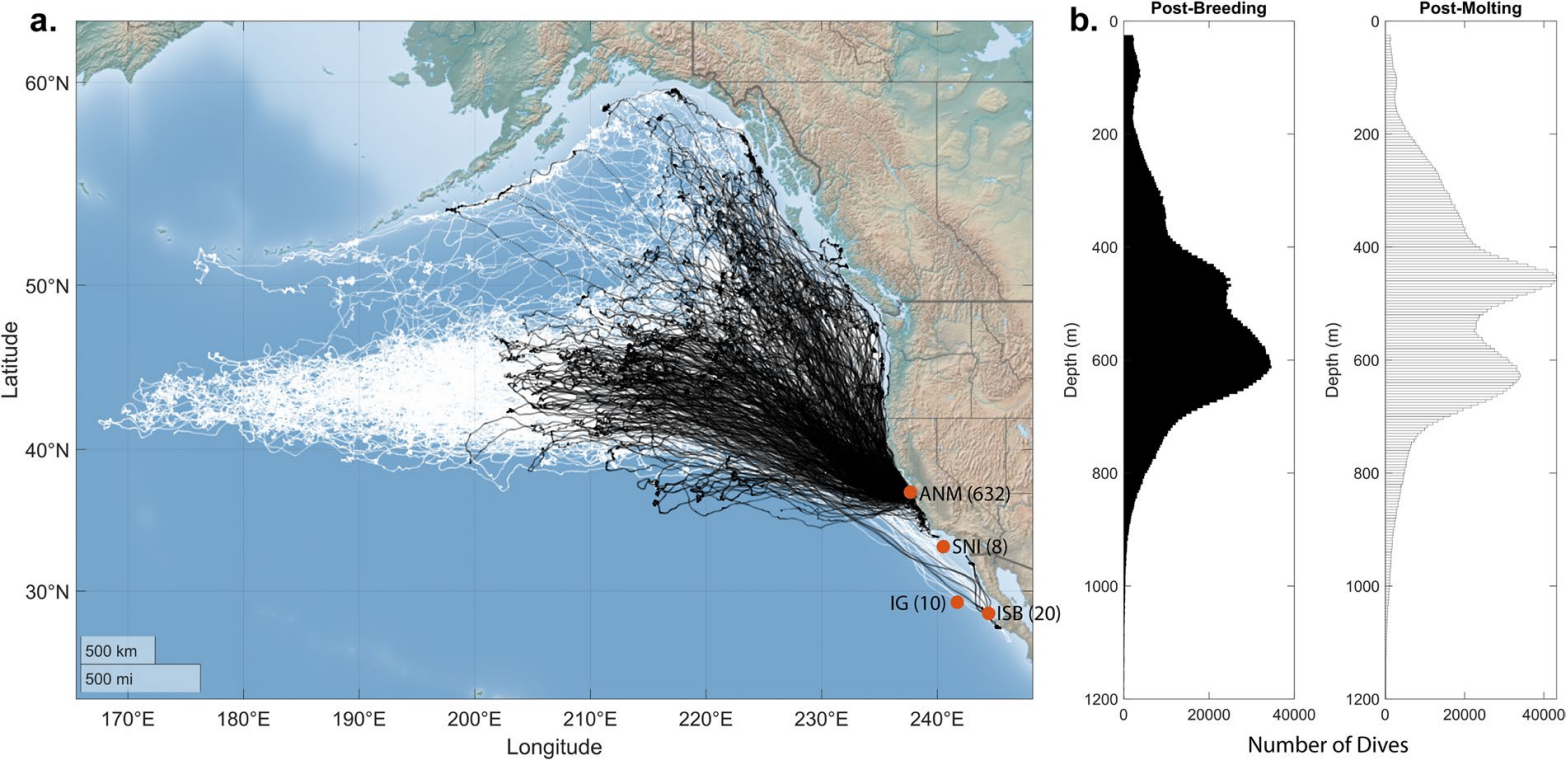
Geographic locations and dive depths from adult female northern elephant seals tracked from 2004 – 2020. (**A**) Locations (Mercator projection) of post-molting (white) and post-breeding (black) elephant seals, data quality 3 or higher (N = 566; see Table 3 for Quality Control (QC) definitions). The four colonies where seals were instrumented, and the number of seals handled at each are indicated (ANM: Año Nuevo Mainland; SNI: San Nicolas Island; IG: Isla Guadalupe; ISB: Isla San Benito). (**B**) Distribution of depths of dives up to 1200 m deep from post-breeding (left) and post-molting (right) TDRs (Time-Depth Records) of quality 3 or better (N = 507). Dives deeper than 1200 m are unusual (~0.05% of dives) and were excluded from this figure. All data shown have been processed as described in Methods.

Little was known about northern elephant seals’ movement and diving behavior before the development of modern-day satellite tags and time-depth recorders (TDRs). TDRs were the first biologgers designed for marine animals^33^ to record how deep and long they dive. Early tags were large and collected relatively small amounts of analog data, which required manual transcription and data processing, limiting their widespread use. It was clear from early tag deployments that elephant seals were deep divers, reaching depths of up to 630 m^34^. Today, digital TDRs are commonly deployed to record depth (via a pressure sensor) and time at consistent sampling intervals (often between 0.125–1 Hz). With today’s memory and battery capabilities, a TDR can provide a high-resolution dive record over multiple months. However, these tags did not provide location data. Deployments of geolocator tags, where coarse locations are inferred based on light levels, revealed extensive use of most of the Eastern North Pacific Ocean, contrasting with boat-based and aerial surveys that suggested that their range was limited to the EEZs of the USA, Canada, and Mexico^8^. Under the auspices of the Census of Marine Life, the Tagging of Pacific Predators (TOPP) program was developed in 2002 with the primary goal of advancing the study of tagging and tracking animal movement^35^. TOPP accomplished this by deploying 4,306 electronic tags, which resulted in 1,791 individual animal tracks from 23 species, covering 265,386 animal tracking days^36^. With support from the TOPP program, large-scale deployments of satellite tags and TDRs to track location and diving behavior began on adult female elephant seals at Año Nuevo in 2004. While the Census of Marine Life ended in 2010, a patchwork of funding has allowed continued deployments of tags on female elephant seals through the present (Fig. 1).

While much remains to be learned about northern elephant seals, the long-term biologging data set that began during the TOPP program has contributed greatly to our understanding of elephant seal behavior, physiology, and ecology. We now know that adult female elephant seals can dive as deep as 1,764 m, nearly triple what the earliest records revealed^37^. The extensive nature of this data set has allowed for expansion beyond “typical” adult female at-sea behavior, which primarily consists of pelagic deep diving at the boundary between the sub-arctic and subtropical gyres of the North Pacific, thousands of kilometers from terrestrial rookeries^1^. For example, we have learned that adult female northern elephant seals also forage on seamounts^38^ and in coastal areas^10^, occasionally remaining within a few days’ swim of breeding colonies. This large elephant seal movement data set has been used to develop and evaluate analytical methods for marine biologging studies^2,39^. For example, the ability to robustly quantify behavior has also allowed for the quantification of at-sea sleep^40^ and the identification of atypical behavior, such as during illness^12^ or in the absence of pregnancy^41^. As the data set covers all years spanning two decades, these data also contribute to a broader understanding of the impacts of a changing climate on marine predator populations^18,22,42,43^.

Data accessibility is increasingly recognized as critical but often lacking for users outside of the research groups responsible for data curation^44–46^. Highly derived subsets of the location data can be accessed through other platforms such as Movebank^47^, the Animal Telemetry Network (ATN)^48^, and the TOPP data set^36^. However, this is the first time all available tracking and diving data have been systematically processed using updated methods, quality controlled, and provided together at full resolution, along with all the processing code. To date, 65 publications have used portions of these biologging data (see S2 for a list of titles and DOIs). Our aim here is to ensure that this northern elephant seal movement data set is Findable, Accessible, Interoperable, and Reusable (FAIR)^49^. All data from 2004 to 2020 that can be found on other platforms are included here, with the addition of diving data and extensive metadata. As with any long-term data set, there are many nuances to understanding the data relative to the natural history of the animal and biases in data collection. For this reason, we encourage potential data users to contact the corresponding authors for guidance.

## Methods

### Animal handling

All animal handling was conducted under National Marine Fisheries Service permit #’s 786–1463, 87–143, 14636, 19108, and 23188, Dirección General de Vida Silvestre permits NÚMS/SGPA/DGVS/05734-2004, NÚMS/SGPA/DGVS/05321-2005, and NÚMS/SGPA/DGVS/03486/17-2017, and with the approval and oversight of the UC Santa Cruz Institutional Animal Care and Use Committee.

Adult female northern elephant seals were sedated for tag attachment and tag recovery using a hand-delivered intramuscular injection of Telazol© (50 mg/mL tiletamine HCl and 50 mg/mL zolazepam HCl) at ~1 mg/kg. Seal mass for dosage was approximated from a visual assessment. Sedation was maintained with intravenous augmentation doses of Telazol (0.15–0.25 mg/kg), ketamine (0.2–0.6 mg/kg), and/or diazepam (0.005–0.025 mg/kg) as appropriate.

**Fig. 2:**
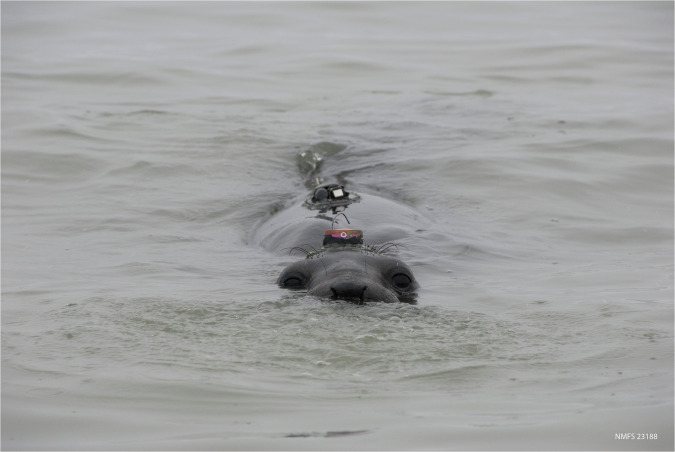
An adult female northern elephant seal carrying a Wildlife Computers Mk-10 satellite tag on her head, and a Mk-9 time-depth recorder paired with a VHF tag on her back. Photo credit: D. Costa.

Tags were attached to the pelage on the animal’s head, back, or jaw using Loctite© 5-minute epoxy. Tags were packaged for attachment using self-amalgamating tape, neoprene, or duct tape to allow easy removal when animals returned from sea. These packaged tags were attached to nylon mesh (¼” heavy delta netting with fish black coating; Memphis Net and Twine Co., Memphis, TN, USA) with zip ties and/or fishing line to increase the contact surface area adhered to the pelage, ensuring that tags remained firmly attached for up to 9 months (Fig. 2). Tags were manufactured by Wildlife Computers (Redmond, Washington, USA), Sea Mammal Research Unit (SMRU) Instrumentation (St. Andrews, Scotland), or Little Leonardo Corporation (Tokyo, Japan) (Table 1).

**Table 1:** The tag models used in this data set, data types collected, typical attachment locations on an elephant seal, frontal cross-section area (cm^2^), mass(g), and manufacturer of each tag model.

Model	Argos	GPS	Depth	TDR Sampling Interval	Attachment Location	Frontal Cross-Section (cm^2^)	Mass (g)	Manufacturer
SPOT	X				Head	1350	110	Wildlife Computers
Mk-10	X	X	X	1, 2, 4, or 8 sec	Head	1767	185	Wildlife Computers
Mk-9			X	1, 2, 4, or 8 sec	Back	342	38	Wildlife Computers
CTD	X		X	2 or 4 sec	Head	2800	370	SMRU
CTD/F	X		X	4 sec	Head	4000	680	SMRU
Kami Kami			X	5 sec	Jaw	320	48	Little Leonardo
Stroke			X	5 sec	Back	320	68	Little Leonardo

Elephant seals typically surface with only their head leaving the water, so satellite tracking tags (i.e., Argos or GPS) are attached to the top of their head to maximize the frequency of location acquisitions (Fig. 2). Some satellite tracking tags (e.g., SMRU CTD-SRDL and Wildlife Computers Mk-10) have integrated pressure and temperature sensors that provide continuous dive and temperature records. When tracking tags without integrated depth sensors were used, a separate TDR (e.g., Wildlife Computers Mk-9) was attached to the animal’s back between the axilla and sternum. A VHF (very high frequency) radio transmitter (e.g., Advanced Telemetry Systems MM200) was also attached to the back to help locate individuals when they returned to land, usually at crowded colonies. In addition, specialized data loggers, such as accelerometers, were periodically deployed. Often, these tags also had integrated pressure sensors and provided a second or third TDR record. Table 1 lists all instrument models used, data types collected, and typical attachment locations for this data set.

Between 7 and 25 individual adult female northern elephant seals were instrumented during each deployment season (post-breeding and post-molting) across all years from 2004–2020 at Año Nuevo Reserve's mainland colony (ANM; California, USA) totaling 652 unique deployments. Additional deployments were completed at Isla San Benito (ISB; Baja California, México) during post-molting 2005 and post-breeding 2006, at San Nicolas Island (SNI; California, USA) during post-molting 2015, and at Isla Guadalupe (IG; Baja California, México) during post-molting 2017 and post-breeding 2018. Tags were recovered when animals returned to shore (and ~5 days post-parturition for pregnant individuals). Recapture rates were 89.1% for post-breeding trips and 81.8% for post-molting trips, totaling 561 recoveries. Instrument malfunctions and unexpected tag losses reduced the data acquisition to 235 post-breeding trips and 224 post-molting trips (75.6% and 65.7% of deployments, respectively), for both high quality diving and tracking data. Total sample sizes, broken down by year and data type, are in Table 2.

**Table 2:** Total number of adult female northern elephant seals deployed and recovered at all colonies and the number of resulting data records with data quality 3 or better (see Table 3 for QC definitions).

Year	Post-Breeding					Post-Molting				
	Deployments	Recoveries	Tracks	TDRs	Tracks + TDRs	Deployments	Recoveries	Tracks	TDRs	Tracks + TDRs
2004	7	6	6	4	4	25	23	18	19	14
2005	19	18	18	18	17	25	23	23	20	18
2005 ISB	—	—	—	—	—	11	9	11	9	9
2006	20	17	18	17	15	24	20	19	17	14
2006 ISB	9	9	4	8	4	—	—	—	—	—
2007	20	17	19	16	16	21	20	20	20	20
2008	23	22	22	22	22	20	14	16	13	10
2009	23	20	20	18	16	8	7	6	7	6
2010	24	23	23	22	21	21	18	16	17	14
2011	21	21	20	18	18	20	15	20	15	15
2012	20	19	18	17	15	22	16	19	16	13
2013	21	15	19	14	13	22	17	17	17	15
2014	20	19	20	17	17	21	15	19	14	13
2015	20	18	18	17	16	20	16	18	15	14
2015 SNI	—	—	—	—	—	8	7	8	6	6
2016	20	16	18	16	15	20	15	19	13	12
2017	9	9	9	9	9	10	8	10	6	6
2017 IG	—	—	—	—	—	6	3	3	3	1
2018	9	9	8	8	7	14	11	14	11	11
2018 IG	4	0	1	0	0	—	—	—	—	—
2019	10	8	9	7	6	11	11	8	7	6
2020	12	11	7	6	4	12	11	9	8	7
Total	311	277	277	254	235	341	279	293	253	224

TDRs (Time-Depth Records) require tag recovery, whereas Argos tracking data are available remotely. Because of various types of tag failure, we do not always have paired tracking and diving data even when tags were recovered, so the number of paired records is also specified (Tracks + TDRs).

### Data processing

**Fig. 3:**
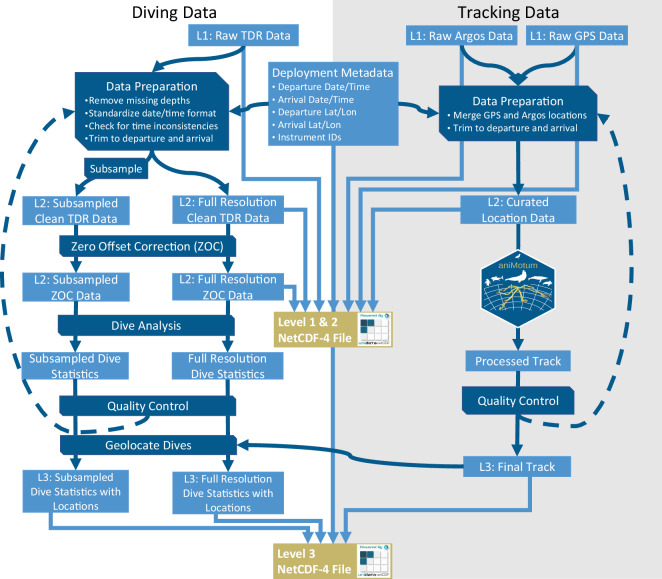
The workflow for processing both tracking and diving data. The grey-shaded section encompasses the processing steps for tracking data and the white section encompasses the processing steps for diving data. Dark blue arrows and boxes indicate processing steps and workflow. Light blue boxes indicate the data type at each processing step, including data level (L1, L2, L3). Light blue arrows show which data components are retained in the final netCDF files for each deployment.

Tracking and diving data were processed and archived following the guidance of Sequeira *et al*.^44^. We include Levels 1 (decoded), 2 (curated), and 3 (interpolated) data for each deployment^44^ for tracking and diving data. The processing workflow for tracking and diving data is depicted in Fig. 3. Additional details of each processing step (dark blue boxes and arrows) are provided in the following paragraphs and all associated code is available (see Code Availability section below). Data preparation, processing, and quality control were completed with custom code written in MATLAB (R2023a^50^) and R (4.2.1^51^) and incorporated functions from the IKNOS toolbox and the package aniMotum (1–1.04^52–55^). Data are saved in netCDF-4 (Network Common Data Format, developed by UCAR/Unidata 10.5065/D6H70CW6) files produced in MATLAB. For each deployment, two netCDF files were created, one containing Level 1&2 data and a second containing Level 3 data (additional details on file structure are in the Data Records section below).

The date and time when animals departed and arrived at the colony were critical for accurately trimming location and diving data to the time the animal spent at sea. These dates were determined, in order of the preferred method, using (1) visual inspection of the TDR record for the start/termination of continuous diving, (2) visual inspection of un-interpolated tracking data for persistent locations corresponding to the colony, or (3) sightings (visual or via VHF scanning) of the seal at the colony by an observer. In very few cases, animals returned to land for less than two days mid-trip before returning to sea. These records have gaps in the diving data corresponding to the time spent on shore.

### Tracking data. 

#### Argos data background

The Argos system uses a constellation of polar-orbiting satellites to geolocate and collect data from platform transmitter terminals (PTTs), including those carried by wildlife. It utilizes the Doppler shift on message frequencies transmitted by the PTTs received by an Argos satellite; subsequent processing of all frequency measurements generates location estimates. This process used a least-squares (LS) filter from Argos’ inception in 1978 until 2011, when the filter was replaced by a multiple-model Kalman filter that reduced location error^56–58^. Argos-based location estimates are assigned a quality class: 3 (LS error radius < 250 m), 2 (250–500 m), 1 (500–1500 m), 0 (>1500 m), A (3 messages, no accuracy for LS filter), B (2 messages, no accuracy for LS filter), Z (for LS: failed to converge)^56^. Error ellipses were introduced for all location classes with multiple-model Kalman filtering, but data included here that were collected prior to 2011 did not undergo multiple-model Kalman filtering.

At typical northern elephant seal movement latitudes (35–60°N), there are currently ~30 Argos satellite passes per day, with each pass lasting between 4 and 13 minutes (https://argos-system.cls.fr/). The seals are at the surface for an average of 2–3 minutes between dives that last 20–40 minutes. Tags were programmed such that Argos transmissions occurred throughout the 24-hour day. We usually received 8–10 locations from an individual seal per 24-hour period.

#### FastLoc GPS background

The FastLoc system was developed by Wildtrack Telemetry Systems Ltd, Leeds, U.K.^59^. It can acquire GPS satellite signals within milliseconds. FastLoc GPS generates pseudo-ranges that are then post-processed to generate location estimates. This rapid acquisition overcomes the limitations of traditional GPS, which often requires too much time to download information from satellites to be useful for tracking aquatic animals that spend brief periods at the surface^58,60,61^. FastLoc GPS location estimates are accurate to 50 m, while Argos locations often have an error of 1–3 km when collected on elephant seals^58^.

#### Data preparation

Location estimates (Level 1 data) were provided by Argos (latitude, longitude, location quality classes, error ellipses if available) or GPS (pseudo-ranges collected by the instrument and solved by the manufacturer’s software to generate latitude and longitude points). Custom-written code in MATLAB imported various formats and created uniform data frames. As seals may have carried Argos and GPS-capable tags, all available location estimates for each deployment were merged into a single file. We removed locations before the seal left the colony and after the seal arrived on shore at the end of the foraging trip. We added a start and end location to the track at the colony where tags were deployed and recovered (when applicable). If location data were collected within 5 days of the seal’s return to shore, we added an end location at the colony. For longer gaps between the last location estimate and the return to shore, the track ends with the last location at sea. This avoided over-interpolating a hypothesized trajectory when a tag’s depleted power supply or other malfunction left a large portion of the seal’s trajectory unknown. The start and end points were assigned a location class of “G,” the same as GPS data, to indicate higher confidence than Argos-based location estimates. These curated data represent Level 2.

#### Data processing

Using bathymetry data, we removed location estimates identified as being on land (Smith & Sandwell v11.1 Topography, data set ID usgsCeSS111 in NOAA’s ERDDAP). We then used the aniMotum package (v1-1.04^52–55^) in R (4.2.1^51^) to generate interpolated locations every three hours using a correlated random walk model and imposing a maximum speed filter of 3 m/s^1,58^. We retained model-determined standard error for each interpolated location estimate for quality control. These interpolated locations are Level 3 data (Fig. 1).

### Diving data. 

#### Data preparation

TDR records underwent manufacturer-specific preparatory steps to convert raw data files into a consistent format for further processing. Wildlife Computers tags’.wch files were decoded into comma-separated value (CSV) format using the manufacturer’s online portal. SMRU tags’ .txt files were imported using custom MATLAB code; lines of text indicating “haulout” periods caused by the tag being dry during a surface interval while the animal was still at sea were removed, and the resulting time-depth series was exported as a CSV. Little Leonardo tags’ .txt files were imported using custom MATLAB code, timestamps were assigned to depth data based on instrument start time and sampling frequency, and the TDR record was exported as a CSV. These decoded files constitute the Level 1 data (“Raw TDR” in Fig. 3) in the netCDF files.

Each decoded file was checked for anomalies in the time-series. For SMRU tag records, at-sea “haulout” periods were filled (using depths of 0 at the tag’s sampling rate) to produce a consistent time series for further processing. In addition, some individual records were corrected based on visual evaluations or known tag issues. For example, several tags recorded the incorrect year but the correct day, month, and time. In this case, we corrected the timestamps to reflect the correct year for that deployment. Little Leonardo depth records were found to be slightly misaligned in time with other TDR records on the same animal. In these instances, a time offset (including shifting from local time to UTC time; median = 8.00 hr, range = 5.68–14.35 hr) and compression factor (median = 2.47 min, range = −50.00 - 17.48 min) were determined on a case-by-case basis and applied to each Little Leonardo record prior to processing. This adjustment ensured that data from multiple TDRs on the same animal were directly comparable. Records were all truncated to the date and time of the animal’s departure from the beach (Level 2 data: “Clean TDR” in Fig. 3).

#### Subsampled data

Dive records from TDRs were collected throughout the study, but earlier tags had limited onboard storage, requiring lower sampling frequency. Different tags can also be programmed with different sampling rates. Consequently, the sampling frequency of dive records varies across the data set (see Table 1). Depth was most frequently recorded every 4 or 8 sec. Since metrics calculated during dive analysis are sensitive to sampling rate, higher resolution records were subsampled to the lowest sampling frequency (8 sec) whenever possible to generate comparable derived metrics across the entire data set. Both full-resolution and subsampled records were processed and are available in the final deployment file, such as the variables TDR1 and TDR1_8S. Some tags (i.e., Little Leonardo TDRs) recorded depth at 5 sec; these were not subsampled, and only full-resolution data were processed. Both full-resolution sampling frequency and depth sensor resolution are indicated in netCDF files as global attributes.

#### Zero offset correction

**Fig. 4:**
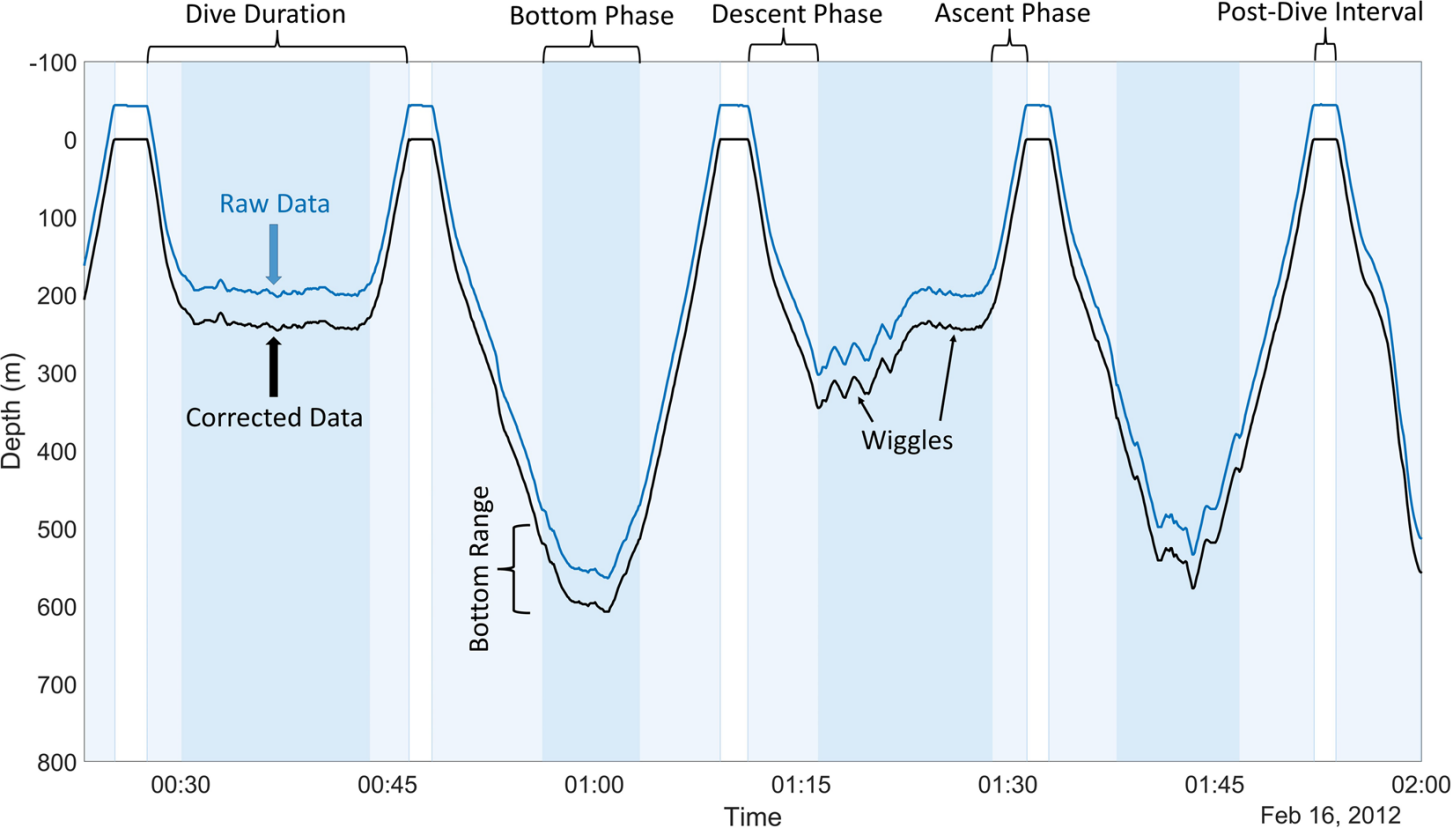
An example of zero-offset correction and dive analysis. The blue line shows raw depth data, and the black line shows corrected data, as described in Methods. Shaded areas indicate the duration of each dive (light shading) and the bottom phase of each dive (darker shading), as determined by the IKNOS dive analysis function.

Level 2 “Clean TDR” data were processed using the dive analysis function from the IKNOS toolbox, which first applies a zero-offset correction (ZOC) to the original depth measurements and exports the depth-corrected TDR data to a CSV file. ZOC is a critical first step in analyzing dive data because it corrects for sensor drift over the length of deployment and provides a clearly defined “surface”^62^. The ZOC function used here considers data within a two-hour window (this time window can be user-defined). It employs a vertical speed filter of 5 m/s to find and correct for spikes in depth sensor readings that are unlikely to be natural vertical movement by the seal. It also trims data outside of a user-defined range of realistic values. The defaults for northern elephant seals were a minimum of −10 m (10 m above the water’s surface) and a maximum of 2,200 m depth. The IKNOS ZOC function is the same method used previously in northern elephant seal studies (i.e., Robinson *et al*.^1^, and others; see S2). However, we additionally checked all records for frequently occurring values “shallower” than −10 m, and assigned a new minimum based on this frequently occurring value (e.g., −40 m). The ZOC algorithm looks for the most repeated value within this two-hour window between the overall minimum depth (usually −10 m) and 15 m to get an approximate surface value for the entire time window. It then identifies more precise surface values for the start and end of each dive. However, these surface values must be within 15 times the instrument depth resolution of the approximate surface value found for the entire time window (e.g., 7.5 m for instrument with 0.5 m resolution). All depth data are corrected by subtracting/adding the offset value that allows the surface to be at 0 m (Fig. 4). The updated depth data are exported alongside the original depth and date/time (data level 2: “ZOC Data” in Fig. 3).

#### Dive analysis

After completing the ZOC, the dive analysis function identifies individual dives and dive phases from the corrected depth data (Fig. 4) and calculates various metrics for each (Level 3 data: “Dive Statistics” in Fig. 3; output as DiveStat.csv). We defined a dive as one with a minimum depth of 25 m and duration of 32 sec. The bottom phase of the dive was determined by identifying inflection points in the descent and ascent rate indicative of a behavioral change. The bottom phase was also constrained to being deeper than 50% of the maximum depth of the dive (Fig. 4). The dive analysis function identifies and quantifies additional characteristics of the dive (e.g., wiggles and bottom range), which may be related to foraging behavior^63^. A complete description of calculated metrics is provided in S1.

#### Geolocating dives

After dive statistic outputs were created, each dive was geolocated along the aniMotum-generated track output (Level 3 tracking data). We linearly interpolated between the two latitudes/longitudes (and corresponding errors) nearest in time to the dive’s start time to generate a starting location and location error for each dive. Once a location was determined, we calculated solar elevation at the start of each dive using the MATLAB function SolarAzEl^64^. The resulting record with combined dive statistics, locations, and solar elevation were then written to netCDF files as individual variables for each metric (Level 3 data).

## Data Records

The adult female northern elephant seal tracking and diving data set is available as netCDF files in two Dryad repositories, one for Level 1&2 data^65^ and one for Level 3 data^66^. We preserved the data in two repositories to increase accessibility and ease of use, as most users will be interested in the Level 3 data, which totals only 2.5GB of storage space. In contrast, the full dive records in the RawCurated.nc files total about 130GB. Two netCDF-4 files were created for each deployment following Sequeira *et al*.^44^ guidelines. The first file (‘*_TrackTDR_RawCurated.nc’) includes Levels 1 and 2 (decoded and curated data) tracking and diving data. The second file (‘*_TrackTDR_Processed.nc’) contains Level 3 data (interpolated data). Both files contain all the deployment and data processing metadata (see S3 for a detailed list and description of all metadata). In addition to the netCDF files, each repository includes a CSV file with the metadata for the dataset (e.g., instrument information, deployment times and locations, and data quality for each deployment) to help the user determine what data are available or appropriate for their question prior to accessing all the netCDF files.

We used a unique deployment identifier (TOPPID) developed for the TOPP project^35^ to link animal and instrument data throughout our workflow. The TOPPID is the start of the name of each netCDF file. The TOPPID is a seven-digit number, for example, 2004001, where the first two digits designate the species (20 is northern elephant seal), digits 3-4 indicate the year (04 is 2004), and digits 5–7 are the deployment serial number (001 is the first deployment for a given year and species). Individual animals were frequently instrumented more than once. Therefore, multiple sets of netCDF files may exist for a single individual. To facilitate the association of deployments to an individual, each netCDF file includes the TOPPIDs for all deployments of that individual as well as a unique seal identifier (Animal_ID).

**Fig. 5:**
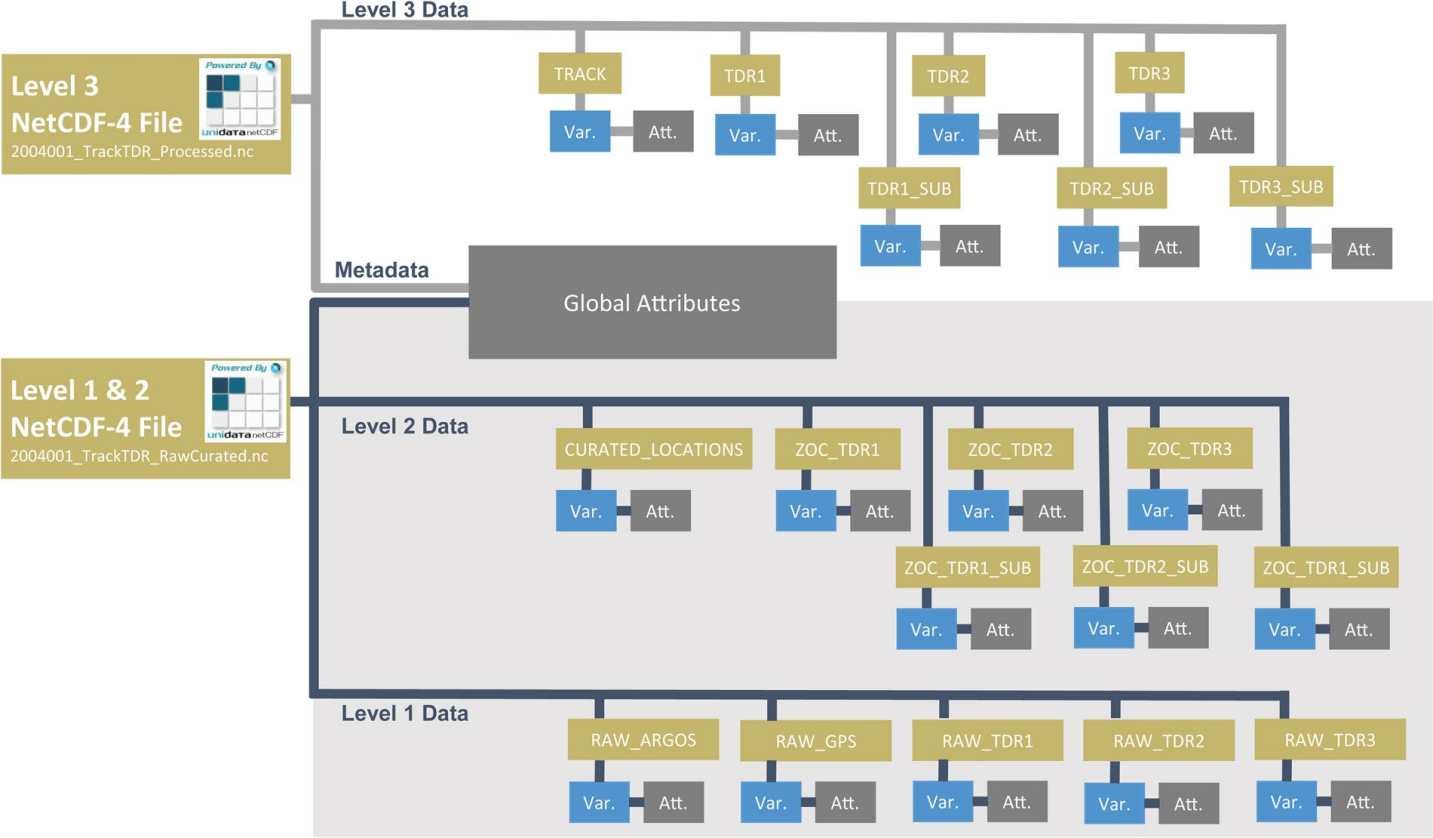
Organizational schematic of the structure of netCDF-4 files used for the northern elephant seal tracking and diving data set. Gold boxes indicate groups created to organize data. Each group contains data stored as variables (blue boxes) with associated attributes (grey boxes). Global attributes contain all metadata for the file, including software versions, data source and ownership, usage instructions, animal information, data quality information, and tag information. This schematic includes most of the preserved data (light blue boxes) illustrated in Fig. 3. Clean TDR data is also included in Level 2 data, but not shown here. A complete list of groups and variable names can be found in the SI.

We utilized the group structure within netCDF-4 to further organize the data provided in each file (Fig. 5, S4, & S5). For example, in the ‘*_TrackTDR_Processed.nc’ files, the aniMotum interpolated track is stored as variables nested within the group “TRACK.” The user would read in the variables TRACK/LAT, TRACK/LON, and TRACK/DATE to create a map for that deployment. Similarly, diving data are stored in separate groups for each TDR instrument deployed on the animal and include the location of the start of each dive (e.g., TDR1/DATE, TDR1/MAXDEPTH, TDR1/LAT, TDR1/LON). Global attributes provide the user with information about data sources, software, and versioning in addition to deployment and animal metadata. Quality control flags (Table 3) and comments are also provided in global attributes to further inform the user. Variable-specific attributes provide additional descriptions of each data field, such as units.

**Table 3:** Definitions for QC flags used in netCDF files for both tracking and diving data.

QC Flag	Meaning	Definition - Tracks	# Tracks	Definition - Dive Records	# Dive Records
1	Complete Data	Tracks are complete. No gaps > 24 hrs between locations AND at least 2 locations/day on average.	248	Dive records are complete, with no data gaps, AND no indications of instrument malfunction.	454
2	Mostly Complete Data	Tracking data have one or more data gaps of up to 2 consecutive days; AND at least 2 locations/day on average.	171	Dive records have one or more data gaps of up to 2 days; OR data show minor indications of instrument malfunction.	1
3	Incomplete Data	Tracks have one or more data gaps greater than 2 consecutive days; AND at least 2 locations/day on average across data collection period (i.e., if tag died, not counting those days).	151	Dive records have one or more gaps greater than 2 consecutive days.	52
4	Questionable Data	Tracks have sparse data (<2 locations/day on average).	55	Dive records have substantial indications of instrument failure.	7
5	No Data	No GPS or Argos locations are available.	28	No dive records are available.	139

Tracking data and dive records each receive a separate QC flag. The number of tracks and dive records from the primary TDR (TDR1 in netCDF file structure) in the current data set are shown for each quality level.

Some animals were simultaneously instrumented with up to three TDRs, typically because of varying study needs over the years. All available depth records were processed and included in the netCDF file as TDR1, TDR2, and TDR3. We have prioritized the records so that the best data available (highest quality and most complete record) are always provided as TDR1. If a deployment had multiple TDRs of equal quality, we prioritized Wildlife Computers tags as TDR1 since their devices are the most abundant within the data set. Instrument metadata are all stored within the netCDF file with a clear indication of the source instrument for each record.

## Technical Validation

### Quality control

Quality control flags 1-5 indicate tracking and diving data quality, as defined in Table 3. Each netCDF-4 file includes a Track QC flag and TDR1, TDR2, and TDR3 QC flags to indicate the data’s quality (completeness and reliability). In the records marked Quality 4 (Questionable Data), we included the Level 1 data (raw records) in the netCDF files, but no additional processing was completed.

#### Tracking data QC

All interpolated tracks were visually inspected for unrealistic results. High standard error in latitude and longitude, reported in the Level 3 tracking data within the netCDF files, accompany low confidence interpolated locations. We further refined our tracking data based on our knowledge of elephant seal movement. We plotted raw location estimates and aniMotum-derived locations and visually assessed each track. Extreme outliers in the raw ARGOS/GPS data (Level 1 data) that had not been removed through aniMotum’s processes but were biologically infeasible were manually removed from the Level 2 (curated) data. Subsequently, the track interpolation was rerun to re-create the Level 3 data. Level 3 tracking data were assigned a Quality Control flag based on the data’s completeness and frequency/duration (Table 3).

#### Diving data QC

All dive statistic outputs were visually inspected for unusual patterns (e.g., many dives of extremely short duration or depth for the species, or many deep dives to identical depth) that could be associated with instrument malfunction. If anomalies were detected, the full-resolution record was examined closely for indications of instrument or data transcription errors or potential mistakes with processing steps (e.g., misnamed or duplicate files). Some records appeared reliable until there was an abrupt change in data quality (often associated with large, frequent spikes in depth). In these cases, the Level 2 records were trimmed to exclude the bad-quality data and then were reprocessed to Level 3. The resulting records were marked Quality 3 (“Incomplete Data”). Records that displayed anomalies throughout were marked Quality 4 (“Questionable Data”). TDR record completeness was checked similarly to tracking data as described above.

Dive statistic outputs were coarsely filtered by descent rate, ascent rate, and dive duration for values outside of what would be possible for an animal to achieve (ascent or descent rates greater than 3 m/s and dive duration greater than 150 min). The values for those individual dives were removed from Level 3 data (but retained in all previous levels). Known tag issues prompted this filtering. For example, some SMRU tags would occasionally mis-record the second half of a dive as having depths at or near the surface within an unrealistically short period from being at depth. Occasionally (frequency of 0.2%), dive ends are incorrectly identified mid-dive, resulting in a post-dive interval of 0 sec. In these instances, the dive with a 0-sec post-dive interval was merged with the subsequent dive to correct that division.

## Usage Notes

The data presented here are freely available under the CC0 1.0 (Creative Commons Universal License), with attribution given to this paper and the Dryad repositories^65,66^. We encourage users to reach out to the corresponding authors for richer insight into the data set. This data set is intended to be a discrete repository for the 2004–2020 period on adult female elephant seals. Derived or low-resolution subsets of the location data have been made available through other projects and data portals. We caution users that these are not independent adult female northern elephant seal data sets. This includes the AniBOS/MEOP data portal (https://www.meop.net/database/meop-databases/)^67^, the Animal Tracking Network (ATN) (https://portal.atn.ioos.us/)^48^, Movebank (https://www.movebank.org/cms/movebank-main)^47^, and MegaMove (https://megamove.org/data-portal/)^23,44^. The data provided here are the complete, full-resolution data that underlie other data sources and include harmonized diving data that have not been released previously. The TOPPIDs described in the Data Records section are unique identifiers for each track and are generally available in these other portals to identify overlapping data.

### Sampling biases

Generally, we have been careful to select presumed healthy animals for sedation and instrumentation. Individuals with known site fidelity to the colony were typically selected for animals deployed at Año Nuevo (most tracks). If age was known, it was usually restricted to 4 to 12-year-olds, young and prime-age females that have reproduced at least once. Furthermore, the data reported here spans two decades of work. During this time, different studies prompted additional non-random population sampling. Examples include focusing on one cohort for a year, repeatedly tracking the same individuals (multiple trips in a row or across multiple years), and intentionally selecting previously tracked females who had used a coastal foraging strategy. Lastly, data collected from other sex and age classes indicate differences in habitat use and movement between these demographic groups, so species-wide inferences cannot be made from these data alone. We strongly encourage researchers to evaluate the metadata provided carefully and contact corresponding authors.

## Supplementary information

Supplementry Information

## Data Availability

All the code written for data processing, including IKNOS functions used for zero offset correction and dive analysis, are available at Github (https://github.com/rholser/NES_TrackDive_DataProcessing) and Zenodo^68^. Extensive documentation of functions and scripts is also provided there. In addition, the authors have provided code in Python, R, and MATLAB for basic access to the netCDF files. They should serve as a model to enable users unfamiliar with the format to access the data (https://github.com/rholser/NES-Read-netCDF/).
