# Supplementary material for: Two decades of three-dimensional movement data from adult female northern elephant seals
Source: Sci Data. 2024 Dec 18;11:1357. doi: 10.1038/s41597-024-04084-4 (PMC11655967; doi:10.1038/s41597-024-04084-4)
Supplement: Supplementary file 1 — Supplementry Information [file 41597_2024_4084_MOESM1_ESM.pdf]

S1 - The output variables created by IKNOS’s dive analysis function and a description of what each metric represents, how it was derived, and output units.

DiveStat Parameter	Description
DiveNumber	Serial number of each dive in record (from 1 to XXXXX)
Year	The date and time at the start of each dive, broken out into separate columns for each unit
Month	
Day	
Hour	
Min	
Sec	
JulDate	Matlab serial number for date (datenum) of the starting time of the dive. Datenum uses 01-Jan-0000 as its point of reference
Maxdepth	Maximum recorded depth (m) of dive
Dduration	Elapsed time (in sec) from identified start to end of dive
Botttime	Time (in sec) spent in bottom phase of dive
DescTime	Time (in sec) from surface to start of bottom phase
DescRate	Average speed (m/s) of descent from surface to start of bottom phase
AscTime	Time (in sec) from end of bottom phase to surface
AscRate	Average speed (m/s) of ascent from bottom phase to surface
PDI	Post-dive interval: the surface time (in sec) following the end of the dive
DWigglesDesc	The number of wiggles (changes in vertical direction) during descent phase
DWigglesBott	The number of wiggles (changes in vertical direction) during bottom phase
DWigglesAsc	The number of wiggles (changes in vertical direction) during ascent phase
TotVertDist	Sum of all of the depth changes during the bottom phase of the dive (in m)
BottRange	Difference between max and min depth of the bottom phase (in m)
Efficiency	Ratio of bottom time to dive cycle time (Dduration + PDI)
IDZ	Intra-Depth Zone: a binary (0,1) indicator of whether the max depth of the dive is within 20% of the max depth of the previous dive

S2 – List of publications that used a subset of the tracking and/or diving data included in the data set being published here, organized by year and first author.

Year	First Author	Title	Journal	DOI
2007	Hassrick	Swimming speed and foraging strategies of northern elephant seals	Deep Sea Research Part II: Topical Studies in Oceanography	10.1016/j.dsr2.2006.12.001
2007	Robinson	A comparison of indirect measures of feeding behaviour based on ARGOS tracking data	Deep Sea Research Part II: Topical Studies in Oceanography	10.1016/j.dsr2.2006.11.020
2007	Simmons	Linking foraging behaviour of the northern elephant seal with oceanography and bathymetry at mesoscales	Marine Ecology Progress Series	10.3354/meps07014
2009	Costa	TOPP as a marine life observatory: using electronic tags to monitor the movements, behaviour and habitats of marine vertebrates	Proceedings of OceanObs'09	10.5270/OceanObs09.cwp.19
2009	Kuhn	Time to eat: measurements of feeding behaviour in a large marine predator, the northern elephant seal *Mirounga angustirostris*	Journal of Animal Ecology	10.1111/j.1365-2656.2008.01509.x
2009	Tremblay	A parsimonious approach to modeling animal movement data	PloS one	10.1371/journal.pone.0004711
2010	Costa	Accuracy of ARGOS locations of Pinnipeds at-sea estimated using Fastloc GPS	PloS one	10.1371/journal.pone.0008677
2010	Harris	Dynamics of marine ecosystems: observation and experimentation	Ecosystems and Global Change	10.1093/acprof:oso/9780199558025.003.0006
2010	Hassrick	Condition and mass impact oxygen stores and dive duration in adult female northern elephant seals	Journal of Experimental Biology	10.1242/jeb.037168
2010	Mitani	Three-dimensional resting behaviour of northern elephant seals: drifting like a falling leaf	Biology Letters	10.1098/rsbl.2009.0719
2010	Robinson	Measurements of foraging success in a highly pelagic marine predator, the northern elephant seal	Journal of Animal Ecology	10.1111/j.1365-2656.2010.01735.x
2010	Simmons	Climate-scale hydrographic features related to foraging success in a capital breeder, the northern elephant seal *Mirounga angustirostris*	Endangered Species Research	10.3354/esr00254
2012	Costa	New insights into pelagic migrations: implications for ecology and conservation	Annual Review of Ecology, Evolution, and Systematics	10.1146/annurev-ecolsys-102710-145045
2012	Costa	Seabirds and Marine Mammals	Metabolic Ecology: A Scaling Approach	10.1002/9781119968535
2012	Maxwell	Benthic foraging on seamounts: A specialized foraging behavior in a deep-diving pinniped	Marine Mammal Science	10.1111/j.1748-7692.2011.00527.x
2012	Robinson	Foraging behavior and success of a mesopelagic predator in the northeast Pacific Ocean: insights from a data-rich species, the northern elephant seal	PloS one	10.1371/journal.pone.0036728
2013	Hassrick	Effects of maternal age and mass on foraging behaviour and foraging success in the northern elephant seal	Functional Ecology	10.1111/1365-2435.12108
2013	Hazen	Predicted habitat shifts of Pacific top predators in a changing climate	Nature Climate Change	10.1038/nclimate1686
2013	Maxwell	Cumulative human impacts on marine predators	Nature Communications	10.1038/ncomms3688
2013	Naito	Unravelling the mysteries of a mesopelagic diet: a large apex predator specializes on small prey	Functional Ecology	10.1111/1365-2435.12083
2013	Schick	Estimating resource acquisition and at-sea body condition of a marine predator	Journal of Animal Ecology	10.1111/1365-2656.12102

Year	First Author	Title	Journal	DOI
2014	Adachi	The foraging benefits of being fat in a highly migratory marine mammal	Proceedings of the Royal Society B	10.1098/rspb.2014.2120
2014	Helm	Overview of effects of oil spills on marine mammals	Handbook of Oil Spill Science and Technology	10.1002/9781118989982.ch18
2015	Maresh	Summing the strokes: energy economy in northern elephant seals during large-scale foraging migrations	Movement Ecology	10.1186/s40462-015-0049-2
2015	Peterson	Deep-ocean foraging northern elephant seals bioaccumulate persistent organic pollutants	Science of the Total Environment	10.1016/j.scitotenv.2015.06.097
2015	Peterson	Marine foraging ecology influences mercury bioaccumulation in deep-diving northern elephant seals	Proceedings of the Royal Society B	10.1098/rspb.2015.0710
2016	Adachi	Searching for prey in a three-dimensional environment: hierarchical movements enhance foraging success in northern elephant seals	Functional Ecology	10.1111/1365-2435.12686
2016	Block	Toward a national animal telemetry network for aquatic observations in the United States	Animal Biotelemetry	10.1186/s40317-015-0092-1
2016	Costa	A Bioenergetics Approach to Understanding the Population Consequences of Disturbance: Elephant Seals as a Model System	Effects of Noise on Aquatic Life II	10.1007/978-1-4939-2981-8_19
2016	Saijo	Linking mesopelagic prey abundance and distribution to the foraging behavior of a deep-diving predator, the northern elephant seal	Deep Sea Research Part II: Topical Studies in Oceanography	10.1016/j.dsr2.2016.11.007
2017	Ferraro	Evaluating gain functions in foraging bouts using vertical excursions in northern elephant seals	Animal Behaviour	10.1016/j.anbehav.2017.05.007
2017	Horton	Route Fidelity during Marine Megafauna Migration	Frontiers in Marine Science	10.3389/fmars.2017.00422
2017	Naito	Oxygen minimum zone: An important oceanographic habitat for deep-diving northern elephant seals, *Mirounga angustirostris*	Ecology and Evolution	10.1002/ece3.3202
2018	Abrahms	Climate mediates the success of migration strategies in a marine predator	Ecology Letters	10.1111/ele.12871
2018	Abrahms	Mesoscale activity facilitates energy gain in a top predator	Proceedings of the Royal Society B	10.1098/rspb.2018.1101
2018	Duarte	Sonification of Animal Tracks as an Alternative Representation of Multi-Dimensional Data: A Northern Elephant Seal Example	Frontiers in Marine Science	10.3389/fmars.2018.00128
2018	Goetsch	Energy-Rich Mesopelagic Fishes Revealed as a Critical Prey Resource for a Deep-Diving Predator Using Quantitative Fatty Acid Signature Analysis	Frontiers in Marine Science	10.3389/fmars.2018.00430
2018	Harrison	The political biogeography of migratory marine predators	Nature Ecology and Evolution	10.1038/s41559-018-0646-8
2018	Huckstadt	The extra burden of motherhood: reduced dive duration associated with pregnancy status in a deep-diving mammal, the northern elephant seal	Biology Letters	10.1098/rsbl.2017.0722
2018	Sequeira	Convergence of marine megafauna movement patterns in coastal and open oceans	Proceedings of the National Academy of the Sciences	10.1073/pnas.1716137115
2019	McHuron	What's in a whisker? Disentangling ecological and physiological isotopic signals	Rapid Communications in Mass Spectroscopy	10.1002/rcm.8312
2019	Pirotta	Modeling the functional link between movement, feeding activity, and condition in a marine predator	Behavioral Ecology	10.1093/beheco/ary183
2019	Sequeira	The importance of sample size in marine megafauna tagging studies	Ecological Applications	10.1002/eap.1947

Year	First Author	Title	Journal	DOI
2019	Shafer	Solar Energy Harvesting Assessments from Migrating Northern Elephant Seals	OCEANS 2019 - Marseille	10.1109/OCEANSE.2019.8867278
2020	Jonsen	A continuous-time state-space model for rapid quality control of argos locations from animal-borne tags	Movement Ecology	10.1186/s40462-020-00217-7
2020	Keates	Chlorophyll fluorescence as measured in situ by animal-borne instruments in the northeastern Pacific Ocean	Journal of Marine Systems	10.1016/j.jmarsys.2019.103265
2020	Yoshino	Acceleration-triggered animal-borne videos show a dominance of fish in the diet of female northern elephant seals	Journal of Experimental Biology	10.1242/jeb.212936
2021	Adachi	Forced into an ecological corner: Round-the-clock deep foraging on small prey by elephant seals	Science Advances	10.1126/sciadv.abg3628
2021	Beltran	Lightscapes of fear: How mesopredators balance starvation and predation in the open ocean	Science Advances	10.1126/sciadv.abd9818
2021	Condit	Birth timing after the long feeding migration in northern elephant seals	Marine Mammal Science	10.1111/mms.12896
2021	Kendall-Bar	Visualizing Life in the Deep: A Creative Pipeline for Data-Driven Animations to Facilitate Marine Mammal Research, Outreach, and Conservation	IEEE VIS Arts Program	10.1109/VISAP52981.2021.00007
2022	Adachi	Whiskers as hydrodynamic prey sensors in foraging seals	Proceedings of the National Academy of Sciences	10.1073/pnas.2119502119
2022	Beltran	Elephant seals time their long-distance migrations using a map sense	Curent Biology	10.1016/j.cub.2022.01.031
2022	Conners	Mismatches in scale between highly mobile marine megafauna and marine protected areas	Frontiers in Marine Science	10.3389/fmars.2022.897104
2022	Costa	Reproductive Energetics of Phocids	Ethology and Behavioral Ecology of Phocids	10.1007/978-3-030-88923-4_8
2022	Holser	Extent and Magnitude of Subsurface Anomalies During the Northeast Pacific Blob as Measured by Animal-Borne Sensors	Journal of Geophysical Research: Oceans	10.1029/2021JC018356
2022	Keates	Foraging behavior of a mesopelagic predator, the northern elephant seal, in northeastern Pacific eddies	Deep Sea Research Part I: Oceanographic Research Papers	10.1016/j.dsr.2022.103866
2022	Kienle	Trade-offs between foraging reward and mortality risk drive sex-specific foraging strategies in sexually dimorphic northern elephant seals	Royal Society Open Science	10.1098/rsos.210522
2022	Merkle	Site fidelity as a maladaptive behavior in the Anthropocene	Frontiers in Ecology and the Environment	10.1002/fee.2456
2023	Adachi	Body condition changes at sea: Onboard calculation and telemetry of body density in diving animals	Methods in Ecology and Evolution	10.1111/2041-210X.14089
2023	Holser	Effects of disease on foraging behaviour and success in an individual free-ranging northern elephant seal	Conservation Physiology	10.1093/conphys/coad034
2023	Kendall-Bar	Brain activity of diving seals reveals short sleep cycles at depth	Science	10.1126/science.adf0566
2023	Welch	Impacts of marine heatwaves on top predator distributions are variable but predictable	Nature Communications	10.1038/s41467-023-40849-y
2023	Krawczyk	Trans-oceanic subsurface photovoltaic performance	Progress in Photovoltaics: Research and Applications	10.1002/pip.3744
2023	*Peterson*	*Foraging behavior and age affect maternal transfer of mercury to northern elephant seal pups*	*In Review*	

S3 - List of global attributes describing dataset and file contents. The attributes included are the same for both *Processed.nc and *RawCurated.nc files, except for Data_Citation_Dataset and DOI which are specific to the respective repositories for the two file types. Most of these values are animal- and deployment-specific and are intended to provide the user with the metadata needed to appropriately use the file contents.

Attribute	Contents
File_Creation_Date	
File_MATLAB_Version	
File_R_Version	
File_aniMotum_Version	
File_IKNOS_DA-ZOC_Version	
File_Contents	Brief description of the data contained in the file (specific to file type)
Data_Owner	Daniel Costa
Data_Public	Yes: data can be used freely as long as data owner is properly cited. We strongly recommend reaching out to the data owner or another of the coauthors (D.Crocker, R.Holser, P.Robinson) for additional information about the study system. Offers of co-authorship would be appreciated, especially given the unique natural history of this organism and the considerable effort required to collect these data.
Data_Citation_Paper	
Data_Citation_Paper_DOI	
Data_Citation_Dataset	Costa, Daniel et al. (2023), Northern Elephant Seal Tracking and Diving Data - Processed, Dryad, Dataset, 10.7291/D18D7W
Data_Citation_Dataset_DOI	10.7291/D18D7W
Data_Type	Tracking and diving time-series
Data_Assembly_By	UCSC/Rachel Holser
Data_Timezome	UTC
Animal_ID	Unique animal identifier
Animal_Species	
Animal_Species_CommonName	
Animal_Sex	M/F
Animal_AgeClass	Adult, Juvenile, etc.
Animal_BirthYear	Year of birth if known; breeding season includes Dec – Feb; all animals born in a breeding season are assigned the year that starts Jan 1st.
Animal_HadPup	Y/N for PM animals (gestational trip); N/A for PB animals (pre-implantation)
Animal_OtherDeployments	List of all TOPPIDs (Deployment_ID) for this unique animal
Deployment_ID	TOPPID - Unique trip identifier for this file
Deployment_Year	
Deployment_Trip	PB/PM; post-breeding or post-molting trips
Deployment_InstrumentsRecovered?	Y/N
Deployment_Manipulation?	Y/N; were animals additionally manipulated with the intention of altering behavior?

Attribute	Contents
Deployment_Departure_Location	Four-letter colony code (ANNU – Año Nuevo; GUAD – Isla Guadalupe; GORD – Gorda; PIBL – Piedras Blancas; PTRE – Point Reyes; SABE – Isla San Benito; SEFI – Southeast Farallon Island; SHIS – Shell Island; SNIS – San Nicholas Island; SMIS – San Miguel Island)
Deployment_Departure_Lat	
Deployment_Departure_Lon	
Deployment_Departure_Datetime	UTC
Deployment_Arrival_Location	Four-letter colony code (see above)
Deployment_Arrival_Lat	
Deployment_Arrival_Lon	
Deployment_Arrival_Datetime	UTC
Data_Track_QCFlag	
Data_TDR1_QCFlag	
Data_TDR2_QCFlag	
Data_TDR3_QCFlag	
Data_TDR1_SamplingFrequency_Hz	
Data_TDR1_DepthResolution_m	
Data_TDR2_SamplingFrequency_Hz	
Data_TDR2_DepthResolution_m	
Data_TDR3_SamplingFrequency_Hz	
Data_TDR3_DepthResolution_m	
Tags_SatTag_Manufacturer	
Tags_SatTag_Model	
Tags_SatTag_ID	
Tags_PTT	
Tags_TDR1_Manufacturer	
Tags_TDR1_Model	
Tags_TDR1_ID	
Tags_TDR1_Comments	
Tags_TDR2_Manufacturer	
Tags_TDR2_Model	
Tags_TDR2_ID	
Tags_TDR2_Comments	
Tags_TDR3_Manufacturer	
Tags_TDR3_Model	
Tags_TDR3_ID	
Tags_TDR3_Comments	

S4 - Index of groups and variables contained in level 1&2 (raw and curated data) netCDF files (*_TrackTDR_RawCurated.nc). These files contain the raw and zero-offset corrected full-resolution time-depth records, raw Argos and GPS location estimates, and combined location estimates prepared for aniMotum processing.

Group	Variable	Description	Units
**RAW_ARGOS**	**Group contains raw argos data**		
RAW_ARGOS	PTT	PTT of satellite tag	
RAW_ARGOS	DATE	Date of Argos-based location estimate	UTC
RAW_ARGOS	CLASS	Location class of Argos-based location estimate	
RAW_ARGOS	LAT	Latitude of Argos-based location estimate	decimal degrees
RAW_ARGOS	LON	Longitude of Argos-based location estimate	decimal degrees
RAW_ARGOS	SEMIMAJOR	Semi-major axis of location estimate's error ellipse	
RAW_ARGOS	SEMIMINOR	Semi-minor axis of location estimate's error ellipse	
RAW_ARGOS	EOR	Semi-major axis orientation from north	
**RAW_GPS**	**Group contains raw GPS data**		
RAW_GPS	DATE	Date of GPS-based location estimate	
RAW_GPS	TIME	Time of GPS-based location estimate	UTC
RAW_GPS	NUM_SATELLITES	Number of GPS satellites detected for location estimate	
RAW_GPS	LAT	Latitude of GPS-based location estimate	decimal degrees
RAW_GPS	LON	Longitude of GPS-based location estimate	decimal degrees
**RAW_TDRs**	**Group contains raw time-depth records**		
RAW_TDR1	DATE		
RAW_TDR1	DEPTH	Uncorrected depth	M
RAW_TDR2	DATE		
RAW_TDR2	DEPTH	Uncorrected depth	M
RAW_TDR3	DATE		
RAW_TDR3	DEPTH	Uncorrected depth	M
**CURATED_LOCATIONS**	**Group contains combined Argos and GPS data, trimmed to time at sea**		
CURATED_LOCATIONS	DATE	Date and time of location estimate	UTC
CURATED_LOCATIONS	LAT	Latitude of location estimate	decimal degrees
CURATED_LOCATIONS	LON	Longitude of location estimate	decimal degrees
CURATED_LOCATIONS	LOC_CLASS	Location class of location estimate	
CURATED_LOCATIONS	SEMI_MAJ_AXIS	Semi-major axis of location estimate's error ellipse	

Group	Variable	Description	Units
CURATED_LOCATIONS	SEMI_MIN_AXIS	Semi-minor axis of location estimate's error ellipse	
CURATED_LOCATIONS	ELLIPSE_ORIENTATION	Semi-major axis orientation from north	
**CLEAN_ZOC_TDRs**	**Groups contain uncorrected and zero-offset corrected depth records**		
CLEAN_ZOC_TDR1	DATE	MATLAB serial date	UTC
CLEAN_ZOC_TDR1	CORR_DEPTH	Depth corrected for true surface	m
CLEAN_ZOC_TDR1	DEPTH	Uncorrected depth	m
CLEAN_ZOC_TDR2	DATE	MATLAB serial date	UTC
CLEAN_ZOC_TDR2	CORR_DEPTH	Depth corrected for true surface	m
CLEAN_ZOC_TDR2	DEPTH	Uncorrected depth	m
CLEAN_ZOC_TDR3	DATE	MATLAB serial date	UTC
CLEAN_ZOC_TDR3	CORR_DEPTH	Depth corrected for true surface	m
CLEAN_ZOC_TDR3	DEPTH	Uncorrected depth	m

S5 – Index of groups/variables contained in level 3 (interpolated data) netCDF files (*_TrackTDR_Processed.nc). These files contain the aniMotum processed tracking data and the dive statistics from each TDR the animal carried (up to 3). Dive statistics were calculated for both full records (e.g., TDR1) and depth data subsampled to every 8 seconds the full record had a sampling frequency every 1, 2, or 4 s (TDR1_8S). Sampling frequencies are included in global attributes (see S2).

Group	Variable	Description	Units
**TRACK**	**Group contains the output from aniMotum-processing tracking data**		
TRACK	LAT	AniMotum track latitude	decimal degrees
TRACK	LON	AniMotum track longitude	decimal degrees
TRACK	DATE	Date and time of location estimate	UTC
TRACK	X	AniMotum interpolated location estimate longitude, World Mercator Projection	km
TRACK	Y	AniMotum interpolated location estimate latitude, World Mercator Projection	km
TRACK	X_SE	AniMotum interpolated location estimate standard error in longitude	km
TRACK	Y_SE	AniMotum interpolated location estimate standard error in latitude	km
TRACK	U	AniMotum estimated velocity in x direction	m/s
TRACK	V	AniMotum estimated velocity in y direction	m/s
TRACK	U_SE	AniMotum estimated standard error of velocity in x direction	m/s
TRACK	V_SE	AniMotum estimated standard error of velocity in y direction	m/s
TRACK	S	AniMotum estimated directionless velocity	m/s
**TDR1**	**Group contains dive statistics and locations for the primary TDR at full resolution**		
TDR1	DATE	Date time at start of dive (UTC)	UTC
TDR1	MAXDEPTH	Max depth recorded during dive	m
TDR1	DURATION	Total duration of dive	sec
TDR1	DESC_TIME	Time spent in descent phase	sec
TDR1	BOTT_TIME	Time spent in bottom phase	sec
TDR1	ASC_TIME	Time spent in ascent phase	sec
TDR1	DESC_RATE	Average rate of descent	m/sec
TDR1	ASC_RATE	Average rate of ascent	m/sec
TDR1	PDI	Surface time after dive	sec
TDR1	WIGGLES_DESC	# of vertical inflections during descent	count
TDR1	WIGGLES_BOTT	# of vertical inflections during bottom	count
TDR1	WIGGLES_ASC	# of vertical inflections during ascent	count
TDR1	TOT_VERT_DIST_BOTT	Sum of all depth changes during bottom	m

Group	Variable	Description	Units
TDR1	BOTT_RANGE	Max-min depth of bottom phase	m
TDR1	EFFICIENCY	Ratio of bottom time to dive cycle	
TDR1	IDZ	Binary indicator of whether max depth is within 20% of previous dive's max depth	
TDR1	SOLAR_EL	Angle of sun relative to horizon	degrees
TDR1	LAT	Decimal latitude at start of dive	decimal degrees
TDR1	LON	Decimal longitude at start of dive	decimal degrees
TDR1	LAT_SE_KM	Latitude error	km
TDR1	LON_SE_KM	Longitude error	km
TDR1	YEAR	Year at start of dive	
TDR1	MONTH	Month at start of dive	
TDR1	DAY	Day at start of dive	
TDR1	HOUR	Hour at start of dive	
TDR1	MIN	Minute at start of dive	
TDR1	SEC	Second at start of dive	
**TDR1_8S**	**Group contains dive statistics and locations for primary TDR at 8 s (subsampled or full-res)**		
TDR1_8S	DATE	Date time at start of dive	UTC
TDR1_8S	MAXDEPTH	Max depth recorded during dive	m
TDR1_8S	DURATION	Total duration of dive	sec
TDR1_8S	DESC_TIME	Time spent in descent phase	sec
TDR1_8S	BOTT_TIME	Time spent in bottom phase	sec
TDR1_8S	ASC_TIME	Time spent in ascent phase	sec
TDR1_8S	DESC_RATE	Average rate of descent	m/sec
TDR1_8S	ASC_RATE	Average rate of ascent	m/sec
TDR1_8S	PDI	Surface time after dive	sec
TDR1_8S	WIGGLES_DESC	# of vertical inflections during descent	count
TDR1_8S	WIGGLES_BOTT	# of vertical inflections during bottom	count
TDR1_8S	WIGGLES_ASC	# of vertical inflections during ascent	count
TDR1_8S	TOT_VERT_DIST_BOTT	Sum of all depth changes during bottom	m
TDR1_8S	BOTT_RANGE	Max-min depth of bottom phase	m
TDR1_8S	EFFICIENCY	Ratio of bottom time to dive cycle	
TDR1_8S	IDZ	Binary indicator of whether max depth is within 20% of previous dive's max depth	
TDR1_8S	SOLAR_EL	Angle of sun relative to horizon	degrees
TDR1_8S	LAT	Decimal latitude at start of dive	decimal degrees

Group	Variable	Description	Units
TDR1_8S	LON	Decimal longitude at start of dive	decimal degrees
TDR1_8S	LAT_SE_KM	Latitude error	km
TDR1_8S	LON_SE_KM	Longitude error	km
TDR1_8S	YEAR	Year at start of dive	
TDR1_8S	MONTH	Month at start of dive	
TDR1_8S	DAY	Day at start of dive	
TDR1_8S	HOUR	Hour at start of dive	
TDR1_8S	MIN	Minute at start of dive	
TDR1_8S	SEC	Second at start of dive	
**TDR2**	**Group contains dive statistics and locations for the second TDR at full resolution**		
TDR2	DATE	Date time at start of dive	UTC
TDR2	MAXDEPTH	Max depth recorded during dive	m
TDR2	DURATION	Total duration of dive	sec
TDR2	DESC_TIME	Time spent in descent phase	sec
TDR2	BOTT_TIME	Time spent in bottom phase	sec
TDR2	ASC_TIME	Time spent in ascent phase	sec
TDR2	DESC_RATE	Average rate of descent	m/sec
TDR2	ASC_RATE	Average rate of ascent	m/sec
TDR2	PDI	Surface time after dive	sec
TDR2	WIGGLES_DESC	# of vertical inflections during descent	count
TDR2	WIGGLES_BOTT	# of vertical inflections during bottom	count
TDR2	WIGGLES_ASC	# of vertical inflections during ascent	count
TDR2	TOT_VERT_DIST_BOTT	Sum of all depth changes during bottom	m
TDR2	BOTT_RANGE	Max-min depth of bottom phase	m
TDR2	EFFICIENCY	Ratio of bottom time to dive cycle	
TDR2	IDZ	Binary indicator of whether max depth is within 20% of previous dive's max depth	
TDR2	SOLAR_EL	Angle of sun relative to horizon	degrees
TDR2	LAT	Decimal latitude at start of dive	decimal degrees
TDR2	LON	Decimal longitude at start of dive	decimal degrees
TDR2	LAT_SE_KM	Latitude error	km
TDR2	LON_SE_KM	Longitude error	km
TDR2	YEAR	Year at start of dive	
TDR2	MONTH	Month at start of dive	
TDR2	DAY	Day at start of dive	

Group	Variable	Description	Units
TDR2	HOUR	Hour at start of dive	
TDR2	MIN	Minute at start of dive	
TDR2	SEC	Second at start of dive	
**TDR2_8S**	**Group contains dive statistics and locations for second TDR at 8 s (subsampled or full-res)**		
TDR2_8S	DATE	Date time at start of dive	UTC
TDR2_8S	MAXDEPTH	Max depth recorded during dive	m
TDR2_8S	DURATION	Total duration of dive	sec
TDR2_8S	DESC_TIME	Time spent in descent phase	sec
TDR2_8S	BOTT_TIME	Time spent in bottom phase	sec
TDR2_8S	ASC_TIME	Time spent in ascent phase	sec
TDR2_8S	DESC_RATE	Average rate of descent	m/sec
TDR2_8S	ASC_RATE	Average rate of ascent	m/sec
TDR2_8S	PDI	Surface time after dive	sec
TDR2_8S	WIGGLES_DESC	# of vertical inflections during descent	count
TDR2_8S	WIGGLES_BOTT	# of vertical inflections during bottom	count
TDR2_8S	WIGGLES_ASC	# of vertical inflections during ascent	count
TDR2_8S	TOT_VERT_DIST_BOTT	Sum of all depth changes during bottom	m
TDR2_8S	BOTT_RANGE	Max-min depth of bottom phase	m
TDR2_8S	EFFICIENCY	Ratio of bottom time to dive cycle	
TDR2_8S	IDZ	Binary indicator of whether max depth is within 20% of previous dive's max depth	
TDR2_8S	SOLAR_EL	Angle of sun relative to horizon	degrees
TDR2_8S	LAT	Decimal latitude at start of dive	decimal degrees
TDR2_8S	LON	Decimal longitude at start of dive	decimal degrees
TDR2_8S	LAT_SE_KM	Latitude error	km
TDR2_8S	LON_SE_KM	Longitude error	km
TDR2_8S	YEAR	Year at start of dive	
TDR2_8S	MONTH	Month at start of dive	
TDR2_8S	DAY	Day at start of dive	
TDR2_8S	HOUR	Hour at start of dive	
TDR2_8S	MIN	Minute at start of dive	
TDR2_8S	SEC	Second at start of dive	
**TDR3**	**Group contains dive statistics and locations for the third TDR at full resolution**		
TDR3	DATE	Date time at start of dive	UTC
TDR3	MAXDEPTH	Max depth recorded during dive	m

Group	Variable	Description	Units
TDR3	DURATION	Total duration of dive	sec
TDR3	DESC_TIME	Time spent in descent phase	sec
TDR3	BOTT_TIME	Time spent in bottom phase	sec
TDR3	ASC_TIME	Time spent in ascent phase	sec
TDR3	DESC_RATE	Average rate of descent	m/sec
TDR3	ASC_RATE	Average rate of ascent	m/sec
TDR3	PDI	Surface time after dive	sec
TDR3	WIGGLES_DESC	# of vertical inflections during descent	count
TDR3	WIGGLES_BOTT	# of vertical inflections during bottom	count
TDR3	WIGGLES_ASC	# of vertical inflections during ascent	count
TDR3	TOT_VERT_DIST_BOTT	Sum of all depth changes during bottom	m
TDR3	BOTT_RANGE	Max-min depth of bottom phase	m
TDR3	EFFICIENCY	Ratio of bottom time to dive cycle	
TDR3	IDZ	Binary indicator of whether max depth is within 20% of previous dive's max depth	
TDR3	SOLAR_EL	Angle of sun relative to horizon	degrees
TDR3	LAT	Decimal latitude at start of dive	decimal degrees
TDR3	LON	Decimal longitude at start of dive	decimal degrees
TDR3	LAT_SE_KM	Latitude error	km
TDR3	LON_SE_KM	Longitude error	km
TDR3	YEAR	Year at start of dive	
TDR3	MONTH	Month at start of dive	
TDR3	DAY	Day at start of dive	
TDR3	HOUR	Hour at start of dive	
TDR3	MIN	Minute at start of dive	
TDR3	SEC	Second at start of dive	
**TDR3_8S**	**Group contains dive statistics and locations for the third TDR at 8 s (subsampled or full-res)**		
TDR3_8S	DATE	Date time at start of dive	UTC
TDR3_8S	MAXDEPTH	Max depth recorded during dive	m
TDR3_8S	DURATION	Total duration of dive	sec
TDR3_8S	DESC_TIME	Time spent in descent phase	sec
TDR3_8S	BOTT_TIME	Time spent in bottom phase	sec
TDR3_8S	ASC_TIME	Time spent in ascent phase	sec
TDR3_8S	DESC_RATE	Average rate of descent	m/sec
TDR3_8S	ASC_RATE	Average rate of ascent	m/sec

Group	Variable	Description	Units
TDR3_8S	PDI	Surface time after dive	sec
TDR3_8S	WIGGLES_DESC	# of vertical inflections during descent	count
TDR3_8S	WIGGLES_BOTT	# of vertical inflections during bottom	count
TDR3_8S	WIGGLES_ASC	# of vertical inflections during ascent	count
TDR3_8S	TOT_VERT_DIST_BOTT	Sum of all depth changes during bottom	m
TDR3_8S	BOTT_RANGE	Max-min depth of bottom phase	m
TDR3_8S	EFFICIENCY	Ratio of bottom time to dive cycle	
TDR3_8S	IDZ	Binary indicator of whether max depth is within 20% of previous dive's max depth	
TDR3_8S	SOLAR_EL	Angle of sun relative to horizon	degrees
TDR3_8S	LAT	Decimal latitude at start of dive	decimal degrees
TDR3_8S	LON	Decimal longitude at start of dive	decimal degrees
TDR3_8S	LAT_SE_KM	Latitude error	km
TDR3_8S	LON_SE_KM	Longitude error	km
TDR3_8S	YEAR	Year at start of dive	
TDR3_8S	MONTH	Month at start of dive	
TDR3_8S	DAY	Day at start of dive	
TDR3_8S	HOUR	Hour at start of dive	
TDR3_8S	MIN	Minute at start of dive	
TDR3_8S	SEC	Second at start of dive	
